# Minimally invasive mesohepatectomy for centrally located liver lesions—a case series

**DOI:** 10.1007/s00464-022-09342-3

**Published:** 2022-06-06

**Authors:** Emrullah Birgin, Vanessa Hartwig, Erik Rasbach, Steffen Seyfried, Mohammad Rahbari, Alina Reeg, Sina-Luisa Jentschura, Patrick Téoule, Christoph Reißfelder, Nuh N. Rahbari

**Affiliations:** Department of Surgery, Medical Faculty Mannheim, Universitätsmedizin Mannheim, Heidelberg University, Theodor-Kutzer-Ufer 1-3, 68167 Mannheim, Germany

**Keywords:** Robotic, Primary liver malignancies, Secondary liver malignancies, Central hepatectomy, Case series, Bisectionectomy

## Abstract

**Background:**

Resection of centrally located liver lesions remains a technically demanding procedure. To date, there are limited data on the effectiveness and safety of minimally invasive mesohepatectomy for benign and malignant lesions. It was therefore the objective of this study to evaluate the perioperative outcomes of minimally invasive mesohepatectomy for liver tumors at a tertiary care hospital.

**Methods:**

Consecutive patients who underwent a minimally invasive anatomic mesohepatectomy using a Glissonean pedicle approach from April 2018 to November 2021 were identified from a prospective database. Demographics, operative details, and postoperative outcomes were analyzed using descriptive statistics for continuous and categorical variables.

**Results:**

A total of ten patients were included, of whom five patients had hepatocellular carcinoma, one patient had cholangiocarcinoma, three patients had colorectal liver metastases, and one patient had a hydatid cyst. Two and eight patients underwent robotic-assisted and laparoscopic resections, respectively. The median operative time was 393 min (interquartile range (IQR) 298–573 min). Conversion to laparotomy was required in one case. The median lesion size was 60 mm and all cases had negative resection margins on final histopathological analysis. The median total blood loss was 550 ml (IQR 413–850 ml). One patient had a grade III complication. The median length of stay was 7 days (IQR 5–12 days). Time-to-functional recovery was achieved after a median of 2 days (IQR 1–4 days). There were no readmissions within 90 days after surgery.

**Conclusion:**

Minimally invasive mesohepatectomy is a feasible and safe approach in selected patients with benign and malignant liver lesions.

Surgical treatment of centrally located liver lesions is technically demanding. Traditionally, surgical resection of central liver segments was managed by extended right or left hepatectomy [1]. However, as the main obstacle of extended hepatectomies remains posthepatectomy liver failure, parenchymal-saving procedures to preserve unaffected liver tissue have been increasingly adopted in the last decades [2, 3]. Mesohepatectomy (also referred to as central hepatectomy/bisectionectomy) with resection of Couinaud segments IV, V, and VIII was first described in 1972 as an alternative treatment option for primary and secondary liver malignancies [4]. A recent meta-analysis demonstrated fewer complications following mesohepatectomy compared to extended hepatectomies [5]. Still, mesohepatectomy is considered among the most difficult procedures in the field of liver surgery. The complexity of mesohepatectomy is primarily due to the proximity to hilar structures and the existence of two transection planes with larger transection surface areas.

Advances in surgical techniques have further prompted the use of minimally invasive techniques for liver resections with the benefit of shorter hospital stay, lower perioperative complications, and fewer blood loss [6, 7]. In few case reports and case series, the evidence of using minimally invasive approaches for a mesohepatectomy has been demonstrated recently [8–14]. However, these studies also included non-anatomic resections, variable combinations of resected liver segments, and limited details of surgical techniques as well as oncological follow-up. To date, none of these studies selectively included patients with a formal mesohepatectomy (i.e., resections of the segments IV, V, and VIII). Therefore, the present case series was performed to report our experience in patients with centrally located lesions undergoing a formal mesohepatectomy using minimally invasive liver surgery at a tertiary care institution.

## Methods

### Study population

This case series has been reported in line with the PROCESS guideline [15]. The institutional review board approved this retrospective review of patient charts from a prospectively recorded database (2020–812-AF11). All consecutive patients who underwent minimally invasive liver surgery between April 2018 and November 2021 at the Department of Surgery, University Hospital Mannheim, Heidelberg University, Medical Faculty Mannheim were assessed for eligibility. The recruitment period was restrained to this time interval as we introduced minimally invasive liver resections at our institution in April 2018. We included all patients with formal mesohepatectomy, i.e., anatomic resections of the segments IVa, IVb, V, and VIII. Patients with non-anatomic resections of central liver segments, additional wedge resections or segmentectomies, preoperative portal vein embolization, extrahepatic resections, vascular resections with reconstructions, other segment combinations, or primarily open liver resections were excluded from the analysis.

### Definitions and outcomes

Liver segments were defined according to Couinaud’s classification and the Brisbane nomenclature [16]. The removal of one Couinaud’s segment was defined as anatomic resection. Centrally located lesions were defined as lesions located within Couinaud segments IV, V, or VIII. The following demographic and clinical characteristics were extracted from the database: age, sex, body mass index, American society of anesthesiologists (ASA) score classification, cardiovascular comorbidities, pulmonary comorbidities, renal insufficiency, diabetes, history of smoking, alcohol abuse, presence of liver steatosis or cirrhosis, history of chemotherapy and other oncological treatment, history of abdominal surgery, and liver resections. The updated Charlson comorbidity index was used to classify the disease burden [17–19]. Operative details were retrieved including data on the surgical approach (robotic, laparoscopic), device of parenchymal transection, use of Pringle maneuver, operative time, and blood loss. The difficulty of liver resection was described by the IWATE score, Southampton classification, and IMM score [20–22].

Histopathological details were analyzed by the Department of Pathology, University Hospital Mannheim, Heidelberg University, Medical Faculty Mannheim [23]. Time-to-functional recovery was defined as a common endpoint including pain control with oral medication, solid food intake, no need for intravenous fluids, and independent mobility of the patient. We further assessed data on postoperative outcomes, such as 90 days mortality rate and 90 days unplanned readmission rate. The Clavien–Dindo classification was used to grade the severity of postoperative complications within 90 days of index operation [24]. Clavien–Dindo complications grade III and higher were considered clinically relevant. Posthepatectomy complications were recorded in line with recommendations of the International Study Group of Liver Surgery [25–27].

### Operative technique

**Fig. 1:**
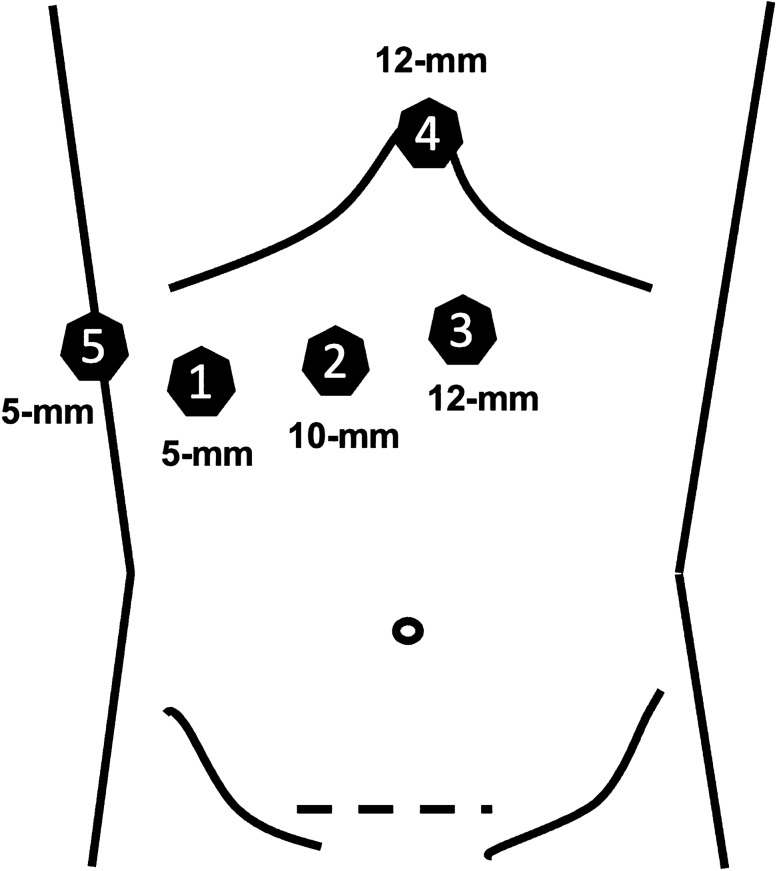
In the laparoscopic approach, the 12 mm ports (No. 3, No. 4) are used for the ultrasound, energy device, linear stapler, or clip applier. The 10 mm port (No. 2) is used for the camera. The 5 mm ports (No. 1, No. 5) are primarily used for graspers, the suction device, or a liver retraction system. The Pfannenstiel incision is used to retrieve the specimen via an extraction bag

**Fig. 2:**
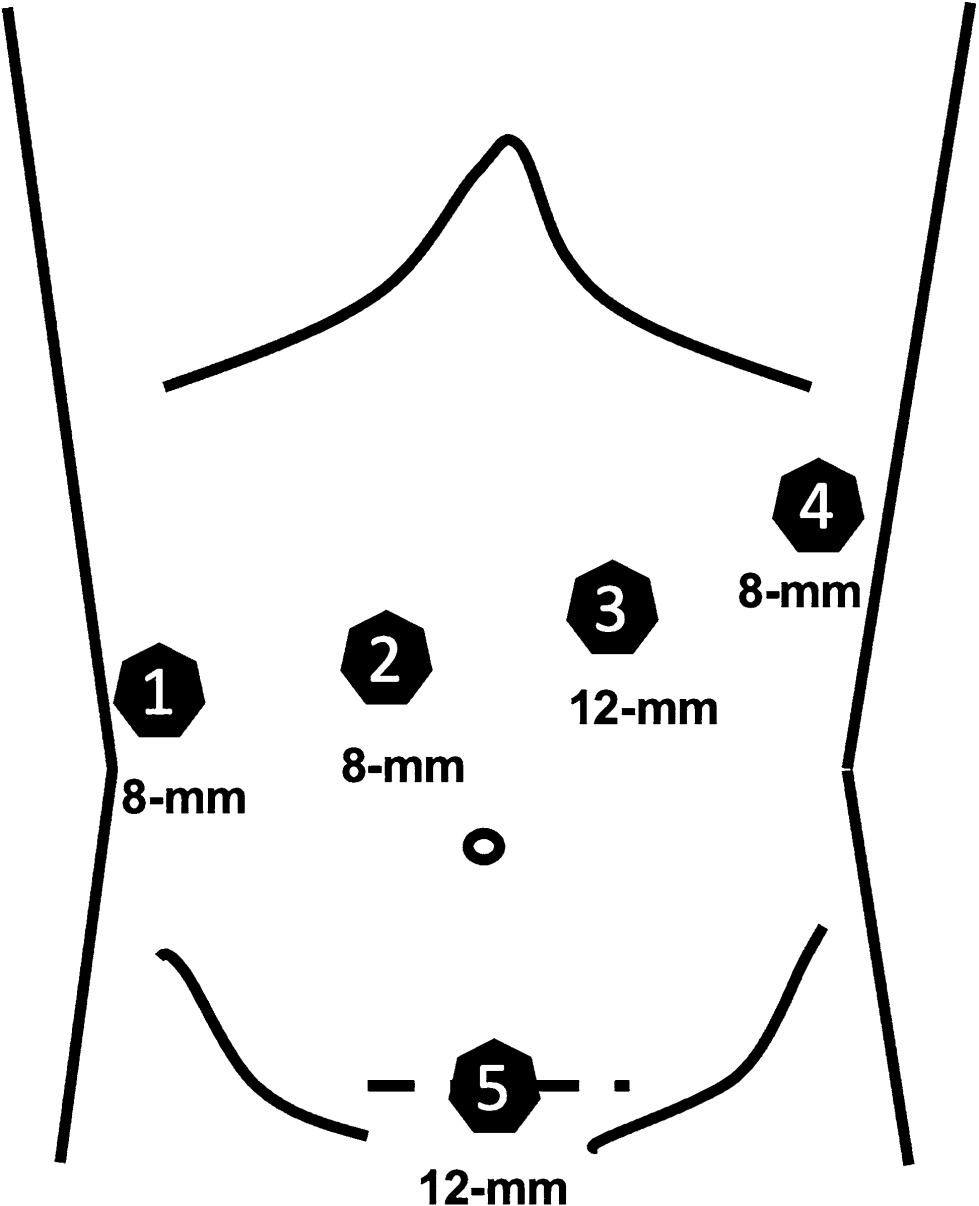
In the robotic-assisted approach, the 8-mm ports (No. 1, No. 2, No. 4) are used for the bipolar forceps, the camera, and the tip-up fenestrated grasper. The 12 mm robotic port (No. 3) is used for the clip applier, linear stapler, and the monopolar scissors (using a port reducer). The 12 mm laparoscopic port is used for the suction device, ultrasound, or clip appliers. The Pfannenstiel incision is used to retrieve the specimen via an extraction bag

Surgical resections were performed by the attending hepatobiliary surgeons with profound expertise in minimally invasive liver surgery. The patient was placed in a supine split-leg French position (reversed Trendelenburg). A pure laparoscopic or robotic-assisted approach was used in all patients with a five-trocar technique (Fig. 1) or four robotic trocars and one laparoscopic trocar technique, respectively (Fig. 2). Robotic trocars were placed at about 12 cm from the rib cage, whereas the laparoscopic trocars were placed close to the rib cage with optimal triangulation to the hilar plate. Pneumoperitoneum was established at 12 mmHg and raised up to 18 mmHg during parenchymal transection. The abdominal cavity was explored visually to rule out extrahepatic disease. Intraoperative ultrasound was carried out to determine resectability and vascular anatomy. An umbilical tape was placed around the portal triad to perform a Pringle maneuver during parenchymal transection. The Glissonean extrahepatic pedicle approach was used, and the portal pedicles were dissected out. The right anterior pedicle was isolated and clamped to visualize the demarcation line between the right anterior and posterior sectors. The Glissonean segment IV pedicles were localized using intraoperative ultrasound and taken down during the course of parenchymal transection along the falciform ligament. The middle hepatic vein was ligated, whereas the right hepatic vein was preserved. A hilar lymphadenectomy was performed for patients with known or suspected cholangiocarcinoma.

The crush–clamp technique in combination with an energy device (LigaSure™, Medtronic, Minneapolis, MN, USA; Thunderbeat™, Olympus Medical Systems Corp., Tokyo, Japan) or robotic scissors (Intuitive Surgical, Sunnyvale, CA, USA) was applied for hepatic transection. Larger intrahepatic vessels, major pedicles, and hepatic veins were divided using linear stapler or Hem-o-lok clips. Specimens were retrieved in an extraction bag through a Pfannenstiel incision.

### Statistics

Categorical variables were presented as frequencies (%) and continuous variables were displayed as median and interquartile range (IQR). The follow-up time was calculated from date of surgery until the date of last follow-up. All analyses were performed using R version 4.0.3 (Vienna, Austria).

## Results

A total of ten patients underwent formal minimally invasive mesohepatectomy during the study period.

The baseline demographics are summarized in Table 1. There were nine men and one woman with a median age of 66 years (IQR 61–76). Patients had a severe disease burden according to the updated Charlson comorbidity index with a median score of six points. The majority of patients had cardiovascular comorbidities (60%). Steatosis was present in 50% of the cases and a total of three patients had Child A cirrhosis. Most patients had previous abdominal surgeries (70%). Primary malignancies were diagnosed in a total of six patients of whom five patients had hepatocellular carcinoma and one patient had cholangiocarcinoma. Of note, another patient was admitted for the treatment of a cholangiocarcinoma in segment IV/V; however, the final pathological result revealed a hydatid cyst. All patients with secondary liver malignancies had colorectal liver metastases and were treated after resection of the primary tumor (rectal (*n* = 2) and sigmoid cancer (*n* = 1)). A total of three patients with hepatocellular carcinoma had a history of previous oncological treatments; one patient had transarterial chemoembolization, one patient had radioembolization with Yttrium-90, and one patient had both transarterial chemoembolization and radioembolization with Yttrium-90. Another patient with colorectal liver metastasis had multiple open wedge liver resections with neoadjuvant chemotherapy (FOLFIRI/Cetuximab) and was admitted for the resection of a recurrent central lesion.

**Table 1:** Baseline demographics

Characteristic	*N* (%) or Median (IQR)
Age, years	66 (61–76)
BMI, kg/m^2^	28 (26–33)
Sex ratio, Male:Female	9:1
ASA	
II	3 (30)
III	7 (70)
Updated charlson comorbidity index (0–24)	6 (5–8)
Cardiovascular comorbidities	6 (60)
Pulmonary comorbidities	3 (30)
Renal insufficiency	2 (20)
Diabetes	4 (40)
Smoking	1 (10)
Alcohol abuse	3 (30)
Steatosis	5 (50)
Liver cirrhosis	3 (30)
Previous abdominal surgery	
Open	4 (40)
Laparoscopic	3 (30)
History of hepatic resection	1 (10)
Diagnosis	
HCC	5 (50)
CCC	1 (10)
CRLM	3 (30)
Hydatid cyst	1 (10)
Previous treatment	
Neoadjuvant chemotherapy	1 (10)
Y90-Radioembolization	2 (20)
TACE	2 (20)

*IQR* interquartile range, *ASA* American society of anesthesiologists, *BMI* body mass index, *HCC* hepatocellular carcinoma, *CCC* cholangiocarcinoma, *CRLM* colorectal liver metastasis, *TACE* transarterial chemoembolization, *Y90* Yttrium-90

Operative details are shown in Table 2. Two patients had robotic-assisted resections while the remaining eight patients were operated using a pure laparoscopic approach. Conversion to open mesohepatectomy was required in one patient due to uncontrollable hemorrhage from the hepatocaval confluence of the right hepatic vein. Parenchymal dissection was performed using energy devices LigaSure™ or Thunderbeat™ in the majority of the patients. The median operative time was 428 min (293–512 min). The median blood loss was 550 ml (413–850 ml). Pringle maneuver for inflow control was applied in a total of six patients with a median occlusion time of 61 min (91–120 min). On final pathology, all specimens had negative resection margins with a median tumor size of 60 mm (37–67 mm).

**Table 2:** Operative details

Characteristic	*N* (%) or Median (IQR)
Surgical approach	
Laparoscopic	8 (80)
Robotic assisted	2 (20)
Parenchymal transection	
Ligasure™	5 (50)
Thunderbeat™	3 (30)
Bipolar forceps (Robot)	2 (20)
Pringle maneuver	
Duration, min	61 (91–120)
Operative time, min	428 (293–512)
Blood loss, ml	550 (413–850)
Tumor size, in mm	60 (37–67)
Number of lesions	1 (1–2)
Difficulty score	
IWATE	10 (11–12)
Southampton classification	9 (8–10)
Institut Mutualiste Montsouris classification	Grade 3

*IQR* interquartile range

Postoperative outcomes are detailed in Table 3. None of the patients developed hemodynamically relevant air embolisms during surgery. The median length of stay was seven days (5–12 days). Time-to-functional recovery was achieved after a median period of two days (1–4 days). Posthepatectomy complications were observed in three patients. These patients had Grade A complications according to the ISGLS classification and required blood transfusion and diuretics. One patient had an intra-abdominal fluid collection and was treated by antibiotics. Severe complications were observed in one patient with a history of multiple open hepatectomies who developed a perforation of his transverse colon after extensive lysis of adhesions during the minimally invasive mesohepatectomy. This patient was taken back to the operating room for creation of a colostomy which was reversed three months after the index operation.

**Table 3:** Postoperative outcomes

Characteristic	*N* (%) or Median (IQR)
Length of stay, d	7 (5–12)
Time-to-functional recovery, d	2 (1–4)
Posthepatectomy complications	
Posthepatectomy liver failure^a^	2 (20)
Posthepatectomy hemorrhage^a^	1 (10)
Specific complications	
Intraabdominal fluid collection	1 (10)
Colon perforation	1 (10)
Postoperative complications^b^	
< Grade III	3 (30)
≥ Grade III	1 (10)
Pathological characteristics of malignant lesions	
Tumor stage	
T1 and 2:T3	3:2^c^/1:0^d^
Nodal status	
N0: N1	1:0^d^
Lymphovascular invasion	
V0:V1	4:1^c^/1:0^d^
L0:L1	5:0^c^/1:0^d^
Resection margin	
R0:R1	5:0^c^/1:0^d^/3:0^e^

*IQR* interquartile range

^a^Grade A International study group of liver surgery

^b^Clavien–Dindo classification

^c^Subgroup of patients with hepatocellular carcinoma (*n* = 5)

^d^Subgroup of patients with cholangiocarcinoma (*n* = 1)

^e^Subgroup of patients with colorectal liver metastasis (*n* = 3)

There was no mortality or unplanned readmission within 90 days after mesohepatectomy. The median postoperative follow-up was 10 (5–21 months). During this period, none of the included patients died. Two patients with hepatocellular carcinoma developed intra- and extrahepatic disease recurrence at 6–8 months after surgery, respectively. Of the three patients who were treated for colorectal liver metastases, none of the patients had signs of recurrent disease at a median of 5 months of follow-up.

## Discussion

The adoption of minimally invasive liver surgery has gained worldwide popularity and even first-level evidence is available for its benefits in comparison to open liver surgery. To date, two randomized multicenter trials found minimally invasive minor hepatectomies to be associated with lower morbidity rates and faster recovery of patients with shorter length of stay, while the oncological results were comparable for both primary and secondary liver malignancies [28, 29]. Still, there are very limited data for complex minimally invasive liver resections of centrally located lesions in the literature. This is mainly due to previous consensus guidelines for laparoscopic surgery which recommended avoiding minimally invasive approaches for centrally located and large lesions in close proximity to hilar structures or major intrahepatic vessels [30, 31]. In 2018, these guidelines were updated and suggested similar perioperative outcomes if advanced expertise in minimally invasive surgery is present [32]. Thereafter, the number of published retrospective cohort studies comparing minimally invasive and open liver resections for centrally located liver lesions increased [12, 33–35]. However, these reports included mixed cohorts of isolated central segmentectomies or any combinations of resected central segments as well as non-anatomic resections. Formal anatomic mesohepatectomies were performed in only 45–65% of these studies providing still limited data on this specific patient cohort [12]. Moreover, these studies included Asian cohorts with distinct clinicopathological features compared to Western cohorts, in particular, a high prevalence of primary liver malignancies. Recently, a multicenter retrospective analysis on robotic versus laparoscopic anterior segmentectomy and central hepatectomy was published. This study presents the largest clinical series of patients with central hepatectomy (*n* = 79) published so far [14]. However, limitations of this combined Eastern and Western experience include a rather long recruitment period of 10 years and inter-institutional differences in perioperative care. In addition, the study provided only limited demographic information, (i.e., no data on comorbidities), surgical details (i.e., whether non-anatomic resections were included or not), and posthepatectomy outcomes (i.e., ISGLS definitions). Therefore, we here describe our experience with minimally invasive formal mesohepatectomy at a Western tertiary care academic center with standardized perioperative care, definitions, and technical details. In the present case series, a total of 10 patients underwent a robotic or laparoscopic mesohepatectomy for both primary and secondary liver malignancies as well as a benign lesion even after intensive pre-treatments and abdominal surgeries. On final pathology, all patients had negative resection margins with an overall uneventful postoperative course. Clinically relevant complications were only observed in one patient who had multiple previous hepatic resections.

In 2014, Conrad et al. reported a clinical series with 32 patients who underwent laparoscopic resections for centrally located lesions in a Western cohort [11]. The postoperative morbidity rate of Grade III and higher complications was 25%. But of the 32 included patients, only a total of six patients had formal mesohepatectomies. Severe complications were detailed in one patient of the mesohepatectomy cohort who developed multiorgan failure due to a small future liver remnant after conversion from a planned mesohepatectomy to an extended right hepatectomy. The average blood loss in their cohort of patients with formal mesohepatectomy was 400 ml with three out of six patients having a blood loss rate exceeding 1000 ml. In our clinical series, we also observed one patient with a severe complication. As opposed to other studies in literature, the majority of our patient cohort had previous abdominal surgeries and, in particular, open hepatic resections as well as high comorbidity burden. Therefore, the operating time and blood loss were as expected higher in our series compared to others, while the time-to-functional recovery remained very short. Several reports exist in the literature that minimally invasive hepatectomy after prior open abdominal surgeries is not associated with a higher morbidity rate compared to open hepatectomy, but a higher rate of adhesions and conversions to open surgery are discussed [36, 37]. The conversion rate of our study was comparable to results in literature for the treatment of centrally located lesions [12, 33–35]. In our study, one patient required a conversion to laparotomy. This patient (BMI 34 kg/m^2^) was previously treated with TACE and Y90-Radioembolization for an HCC and experienced a tear of the right hepatic vein at the hepatocaval confluence during anatomic resection of segment VIII. Of note, the postoperative course of this patient was uneventful, and the patient was discharged on postoperative day six. The decision for conversion was made to control the bleeding. Another potential risk associated with minimally invasive liver resections is gas embolism which has been previously reported of being more frequent in case of high intraperitoneal pressure exceeding 15 mmHg in animal studies [38, 39]. Therefore, many hepatobiliary surgeons use intra-abdominal pressure rates of 10–14 mmHg to control back bleedings from hepatic veins during parenchymal transection, whereas other hepatobiliary surgeons reported pressure rates of 16–20 mmHg without safety issues [30, 40, 41]. However, a definitive consensus on which pressure to be used is not available due to the lack of prospective clinical trials [30, 42]. In our study, none of the patients had gas embolism using a pneumoperitoneum rate up to 18 mmHg. The only patient with a severe complication after surgery had multiple prior open hepatectomies with migration of the transverse colon to the resected liver surfaces. This led to an unintended colon perforation after extensive adhesiolysis with formation of a colostomy. Furthermore, all patients who had specific posthepatectomy complications were managed by conservative treatment only. None of the included patients developed posthepatectomy liver failure or died within 90 days after surgery which is consistent to previous studies [12, 33–35].

Various transection techniques were used in this case series. A recent network meta-analysis demonstrated that bipolar cautery techniques were associated with lower blood loss and operating time in open liver resections; however, the best transection technique in minimally invasive liver surgery still remains unclear [43]. In addition, not all transection techniques of laparoscopic liver surgery are available for robotic-assisted resections. In the present study, two patients underwent robotic-assisted mesohepatectomy. A recent consensus statement on robotic hepatectomy revealed that robotic-assisted resections are safe and feasible, while operative time is longer and blood loss higher than laparoscopic hepatectomy [44]. These findings have been recently confirmed in a propensity-matched analysis of laparoscopic versus robotic right anterior and central hepatectomy which is in line with our experience that robotic approaches strongly facilitate hilar dissection and resections of posteriosuperior segments [14]. This note has also been confirmed recently as difficult resections defined by the IWATE score might benefit from robotic-assisted surgery with respect to postoperative complications [45]. In our series, the procedures were characterized by high difficulty levels as revealed by the applied difficulty scores. We believe that robotic hepatectomy has at least the potential to be an equivalent alternative to laparoscopic and open hepatectomy but in the absence of randomized trials clear indications for robotic-assisted hepatectomy must be determined.

Most patients in the present series had large primary and secondary malignant lesions. The resection margins were all negative, and few patients had neoadjuvant chemotherapy or local ablative treatments. Thus, minimally invasive mesohepatectomy enables appropriate oncological outcomes as revealed by the present case series, although the cohort included heterogeneous malignancies. Unfortunately, long-term survival data of large study cohorts remains rare for mesohepatectomy [6, 46]. Therefore, future prospective trials on the use of minimally invasive major hepatectomy, in particular, robotic-assisted resections are required to assess long-term outcomes, health-related quality of life, and costs. In conclusion, minimally invasive mesohepatectomy is safe and feasible for the treatment of malignant and benign lesions but prospective trials are required to determine final recommendations on its applicability and the selection of patients.
